# An Efficient Incremental Mining Algorithm for Discovering Sequential Pattern in Wireless Sensor Network Environments

**DOI:** 10.3390/s19010029

**Published:** 2018-12-21

**Authors:** Xin Lyu, Hongxu Ma

**Affiliations:** College of Computer and Information, HoHai University, Nanjing 210098, China; mdaha@hhu.edu.cn

**Keywords:** WSNs, big data, incremental mining, prefix projection database, reticular sequence tree

## Abstract

Wireless sensor networks (WSNs) are an important type of network for sensing the environment and collecting information. It can be deployed in almost every type of environment in the real world, providing a reliable and low-cost solution for management. Huge amounts of data are produced from WSNs all the time, and it is significant to process and analyze data effectively to support intelligent decision and management. However, the new characteristics of sensor data, such as rapid growth and frequent updates, bring new challenges to the mining algorithms, especially given the time constraints for intelligent decision-making. In this work, an efficient incremental mining algorithm for discovering sequential pattern (novel incremental algorithm, NIA) is proposed, in order to enhance the efficiency of the whole mining process. First, a reasoned proof is given to demonstrate how to update the frequent sequences incrementally, and the mining space is greatly narrowed based on the proof. Second, an improvement is made on PrefixSpan, which is a classic sequential pattern mining algorithm with a high-complexity recursive process. The improved algorithm, named PrefixSpan+, utilizes a mapping structure to extend the prefixes to sequential patterns, making the mining step more efficient. Third, a fast support number-counting algorithm is presented to choose frequent sequences from the potential frequent sequences. A reticular tree is constructed to store all the potential frequent sequences according to subordinate relations between them, and then the support degree can be efficiently calculated without scanning the original database repeatedly. NIA is compared with various kinds of mining algorithms via intensive experiments on the real monitoring datasets, benchmarking datasets and synthetic datasets from aspects including time cost, sensitivity of factors, and space cost. The results show that NIA performs better than the existed methods.

## 1. Introduction

WSNs are made up of a large number of sensor nodes deployed in the monitored area, and by wirelessly communicating between the nodes, it forms a multi-hop self-organized network system to perceive, collect, and process the information of objects continuously, where the results are then forwarded to the central node. Nowadays, WSNs have been widely used in all sorts of fields, such as smart homes [1], urban traffic [2,3], safety monitoring of large buildings [4,5], environmental monitoring [6,7], space exploration [8], and so on, such that it produces diverse and large amounts of sensor data constantly. Evidently, the data contains enormous potential value, which shall be of great value for decision and management. For instance, in dam safety monitoring, there are at least thousands of sensors that are embedded in the dam or spread over the surrounding area, and they feed back various signals in real time, such as seepage pressure, deformation, displacement, and water level. If the correlation between different kinds of measured values, like the value of displacement and water level, can be discovered, then it is able to set the safety threshold of water level based on the discovered patterns or rules. Furthermore, the safety condition of the dam can be predicted through some forecasting information, like rainfall. Obviously, these are extremely helpful for decision-support, especially in the scenario of emergency response and providing early risk warnings. Therefore, it is necessary to design effective data mining and analysis methods to discover the knowledge buried in the sensor data.

The sensor data commonly belongs to a type of time series, and sequential pattern mining (SPM) is an important and active research topic in data mining [9,10,11,12,13]. The main target of SPM is to discover the set of frequent sequences measured by a user-specified minimum support threshold, which was first proposed by Agrawal et al. in 1995 [11]. It finds customer-purchased sequences to predict whether there is a high probability that, when customer buys some products, they will buy some other products in later transactions. With a certain minimum support specified by the user, the problem of mining sequential patterns is to find the maximal sequences among all sequences satisfying the support constraint. For instance, there is a dataset that contains five sequences: 〈(30)(90)〉,〈(10 20)(30)(40 60 70)〉,〈30 50 70〉,〈(30)(40 70)(90)〉, 〈(90)〉. When minimum support set to 25%, two sequences: 〈(30)(90)〉 and 〈(30)(40 70)〉 are maximal among those satisfying the support constraint, and they are the desired sequential patterns. In Reference [11], three algorithms, AprioriAll, AprioriSome, and Dynamicsome, were presented to solve the problem of SPM, and the performance of these algorithms were evaluated empirically using synthetic data. They are the foundational algorithms of sequence data mining. Since then, many algorithms have been proposed to improve mining time. Zaki [13] proposed a new algorithm named SPADE (Sequential PAttern Discovery using Equivalence classes) for fast mining of sequential patterns, which decomposed the original problem into smaller sub-problems using equivalence classes on frequent sequences. Thus, the mining process is completed in only three database scans. The experiments were conducted both on synthetic datasets and a real dataset to show the advantages of SPADE. Ayres et al. [12] introduced a novel depth-first search strategy with effective pruning mechanisms for mining sequential patterns, and a vertical bitmap representation was utilized to store each sequence in the database for efficient support counting. The proposed algorithm was tested on numerous synthetic datasets, and the results demonstrated that it outperforms SPADE [13] on large datasets by over an order of magnitude. Philippe et al. [10] analyzed the major problems of the existing SPM algorithms, and two novel algorithms, RuleGrowth and TRuleGrowth, were proposed to mine “partially-ordered sequential rules.” RuleGrowth used a pattern-growth approach for discovering valid rules to avoid considering rules not appearing in the database. TRuleGrowth allows the user to specify a sliding-window constraint on rules to be mined, which reduced the execution time and the number of valid rules. The performance study with four real-life datasets showed that the proposed algorithms have excellent performance and scalability compared to the baseline algorithms, and they are suitable for a real application. In particular, Philippe founded an open-source data mining library, called SPMF (Sequential Pattern Mining Framework) [14,15], specialized in pattern mining, offering implementations of 156 data mining algorithms. It has been cited or used in more than 600 publications to solve applied problems from a wide range of domains. Its implementations are also commonly used as benchmarks in research papers. Wu et al. [9] proposed a new a-priori-based sequence pattern mining algorithm named NOSEP (NOnoverlapping SEquence Pattern), in which a nonoverlapping sequence pattern allows sequence letters to be utilized flexibly for pattern discovery; it also used a designed data structure, Nettree, to calculate the exact occurrence of a pattern in the sequence. Sixteen benchmark datasets from three domains in real life were used in the experiments, and the results demonstrated that NOSEP can discover more frequent patterns than state-of-the-art algorithms.

A great part of SPM studies has been focused on the static databases. However, databases utilized in the real world are dynamic with frequent insertions or deletions, such as the monitoring scenario mentioned before. Discovered sequential patterns may become invalid since sequences are changed in dynamic databases. An intuitive way to solve this problem is to rescan the updated database and re-mine the sequential patterns. This requires considerable computations when the database is huge, and it is inefficient in real applications, especially the number of changed sequences is very small. To deal with dynamic databases, various methods were proposed. Cheng et al. proposed an incremental mining algorithm named IncSpan [16], based on PrefixSpan [17]. The frequent patterns can be efficiently discovered via projection mining on semi-frequent sequences and the sub-sequence of frequent sequences. However, Nguyen et al. [18] had proved IncSpan does not display completeness, and presented an improved version named IncSpan+. In order to achieve completeness, the algorithm should carry out projection mining on more sequences, resulting in low efficiency. Liu et al. proposed a sequence tree-based incremental mining algorithm ISPBS (Incremental Sequential Pattern mining Based on Sequence tree) [19]. The mining information is stored in the sequence tree. To obtain the new set of frequent sequence, ISPBS just needs to add the incremental sequences to the sequence tree. However, it apparently has to bear excessive memory consumption to store all the sequence trees. The pre-large concept was also proposed to deal with dynamic database [20,21], which had been proved to be more efficient than the a-priori-like algorithms in batch mode. The pre-large concept employs two thresholds, which are upper and lower, and all the patterns are classified into nine cases. When new data are inserted, just a few cases require rescanning operation. Hence, the methods based on the pre-large concept have better performance than the methods that always rescan the original database, but they still need a number of re-scans and generate many candidate patterns. Lee et al. [22] suggest an efficient pattern mining approach, which is still based on the pre-large concept. It introduces a proper data structure for mining patterns, enabling the mining process to be completed with a one-time scan. Huynh et al. [23] proposed a parallel approach named MCM-SPADE (Multiple threads Co-occurence Map-SPADE) using a multi-core processor system for an SPM with a very large database. The frequent pattern mining expresses item information of databases as a binary form, so it has a limitation that it cannot consider real data’s non-binary character. For instance, in market databases, an item has its own different price or profit and can be sold multiple copies in a transaction, which are not binary data. To address this issue, some recent studies incorporated the concept of utility into classic SPM, leading to the emergence of high utility sequential pattern (HUSP) mining. In HUSP mining, the utility of a sequence represents its importance, which may be measured in terms of profit or other information valuable for users. Wang et al. [24] introduced a tighter upper bound of the utility of a sequence (TSU) and design a novel data structure to maintain high utility sequences, facilitating incremental HUSP mining. Lin et al. [25] proposed a novel high-utility sequential pattern mining with multiple minimum utility thresholds framework. LS-tree (Lexicographic Sequence-tree) and UL-list (Utility Linked-list) structure are designed to efficiently mine HUSPs, and in the meantime, three pruning strategies are introduced to improve the performance of the proposed algorithm. SPM is also becoming important in new types of sensor networks, such as body sensor networks [26]. Through mining the complete set of periodically/regularly occurring patterns in a BSN (Body Sensor Network) data stream, it would be useful to promote and assist important decision-making in healthcare.

Motivated by the real applications and the problems mentioned above, an efficient incremental mining algorithm for discovering sequential pattern named NIA (Novel Incremental Algorithm) is proposed. The main contributions of this paper are as follows: (1) the mining space is greatly narrowed based on the analysis of the sequence-related properties in the updating process; (2) PrefixSpan+, an improvement of the original PrefixSpan is proposed to improve the mining efficiency; and (3) a novel structure called a reticular sequence tree is designed to count the support number quickly. The rest of this paper is organized with the following contents. In Section 2, the related definitions are explained. In Section 3, the integrity proof, the suggested PrefixSpan+, and fast counting algorithm are discussed in detail. In Section 4, the experiments are conducted on real sensor datasets with their analysis results. In the final section, we conclude this paper.

## 2. Definitions

An original sequence database *SDB* is the set of a two-tuple <sid,α>, α denotes sequence and sid is the corresponding serial number. $sup^{SDB}(\alpha )$ denotes the support of sequence α and $count^{SDB}(\alpha )$ (shortened to $c^{SDB}(\alpha )$) is the number of two-tuples that contains α, such that $c^{SDB}(\alpha )=sup^{SDB}(\alpha )\times \left|SDB\right|$. If $sup^{SDB}(\alpha )\geq \min\_\sup$, then α is called a sequential pattern in *SDB*. Correspondingly, the incremental sequence database *sdb* is also the set of <sid,α>, where $sup^{sdb}(\alpha )$ and $c^{sdb}(\alpha )$ are the support of α and the number of two-tuples that contain α, respectively. UD is the updated database that involves all the sequences in *SDB* and *sdb*. $sup^{UD}(\alpha )$ denotes the support of α in UD and $c^{UD}(\alpha )$ denotes the support number. The incremental mining algorithm is to discover the set of frequent sequences in UD.

**Figure 1:**
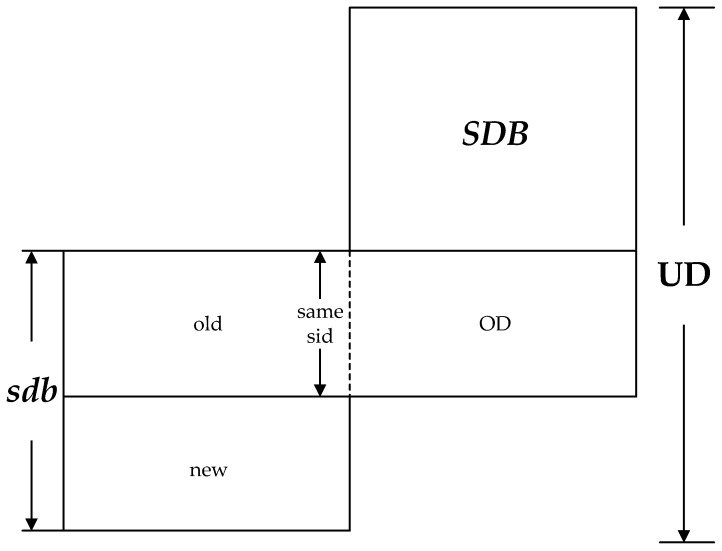
The updated database UD which is the combination of *SDB* and *sdb*.

The updated database UD is the combination of *SDB* and *sdb*. The subset “old” is a subset of *sdb* in which the *sid* of each sequence is same with a certain *sid* appears in *SDB*, and “new” is another subset of *sdb* in which the *sid* of each sequence does not appear in *SDB*. Thus, the number of sequences in UD is equal to |UD|=|SDB|+|sdb|−|old|, such that if old=ϕ, then |UD|=|SDB|+|sdb|; if all the *sid*s in *sdb* have appeared in *SDB*, that is, the set of “new” is empty, then |UD|=|SDB|. Figure 1 shows an updated database UD that is the combination of the sets *SDB* and *sdb*.

OD is a subset of *SDB*, of which the set of *sid* is the same as that used in old. $c^{OD}(\alpha )$ denotes the support number of α in OD. The combination of OD and old is called DD, and $c^{DD}(\alpha )$ denotes the support number of α in DD.

All the symbols used in this paper are listed in Table 1.

Table 2 displays a sequence database (*SDB*) containing six sequences. Suppose the minimum support is min_sup=33%, then the support number of a frequent sequence must be no less than 2, so the set of frequent sequences in *SDB* is {a,b,c,d,ad,ac,bb,ca}.

An increment database *sdb* is shown in Table 3, and the sequence database of the updated *SDB* named UD is shown Table 4. There are nine sequences in UD, so the support of frequent sequence in UD must be no less than 3, according to the minimum support; therefore, some of the frequent sequences in *SDB* are no longer frequent sequences in UD, such as {c,ad,ca}, and {e,be,bd} becomes a frequent sequence in UD.

Table 5 displays an incremental database *sdb* of UD with the sequences (SID=2,4,5,8) that are from the original UD, and the sequences (SID=10,11,12) are the new ones. The updated sequence database, the new UD, is shown in Table 6. The elements with underline denote the elements added to the original sequences. For the same min_sup, the frequent sequences in UD are {a,b,d,f,bd,bf,ad}.

Table 7 displays a sequence database OD, in which the sequences are from *SDB* and their *SID* are same with that in *sdb*.

Table 8 shows a sequence database DD, which is a combination of the sequence databases of old and OD.

## 3. An Efficient Incremental Mining Algorithm for Discovering Sequential Pattern in a WSN Environment

In order to maintain the set of frequent sequences and reduce the searching cost on the original database, a novel incremental mining algorithm for discovering sequential patterns (NIA) is proposed. First, the changes of the potential frequent sequence are discussed regarding when the database is updated, then an algorithm named PrefixSpan+ is suggested to mine the required spaces, that is, the incremental database *sdb* and the projection database on OD, to obtain complete potential frequent sequences. Finally, the sequence support degree is computed. A data structure, named reticular sequence tree, is introduced in this process. The sequence tree is generated using the corresponding potential frequent sequence, then each support degree can be computed from the root. This method greatly reduces the time to scan the original database during computing the sequence support.

### 3.1. PrefixSpan+

PrefixSpan (Prefix-Projected Pattern Growth) [17] is a classic SPM algorithm. Its general ideal is to examine only the prefix subsequences and project only their corresponding postfix subsequences into projected databases. In each projected database, sequential patterns are grown by exploring only local frequent patterns. There is no candidate sequence produced during the mining process and the projected database keep shrinking; however, PrefixSpan still involves a high computation cost owing to constructing the projection database recursively, so it is unsuitable for large-scale sequence datasets with various items. In this section, an improvement of the original PrefixSpan algorithm, called PrefixSpan+, is suggested with the use of a structure named CMAP (Co-occurrence MAP) [27] in order to enhance the efficiency of the recursive process mentioned above. CMAP is a structure for storing co-occurrence information, and it was originally used to candidate pruning for vertical sequential pattern mining. Here, it is used to extend the prefixes to frequent sequential patterns instead of constructing projection databases recursively. The definition of CMAP is given below.

**Definition** **1.** *An item k is said to succeed by i-extension to an item j in a sequence $\langle I_{1},I_{2},\cdots ,I_{n}\rangle$ iff $j,k\in I_{x}$ for an integer x such that 1≤x≤n and k≻jlex, where ${≻}_{lex}$ means lexicographical order.*

**Definition** **2.** *An item k is said to succeed by s-extension to an item j in a sequence $\langle I_{1},I_{2},\cdots ,I_{n}\rangle$ iff $j\in I_{v}$ and $k\in I_{w}$ for some integers v and w such that 1≤v<w≤n.*

**Definition** **3.** *A Co-occurrence map (CMAP) is a structure mapping each item k∈I to a set of items succeeding it. We define two CMAPs named $CMAP_{i}$ and $CMAP_{s}$. $CMAP_{i}$ maps each item k to the set $cm_{i}\left(k\right)$ of all items j∈I succeeding k by i-extension in no less than minsup sequences of SDB. $CMAP_{s}$ maps each item k to the set $cm_{s}\left(k\right)$ of all items j∈I succeeding k by s-extension in no less than minsup sequences of SDB.*

The CMAP structure can be used for pruning candidates generated by sequential pattern mining algorithms based on the following properties.

**Property** **1** **(pruning an *i-extension*).** Let there be a frequent sequential pattern A and an item k. If there exists an item j in the last itemset of A such that k belongs to $cm_{i}\left(j\right)$, then the *i-extension* of A with k is frequent.

**Property** **2** **(pruning an *i-extension*).** Let there be a frequent sequential pattern A and an item k. If there exists an item j∈A such that the item k belongs to $cm_{s}\left(j\right)$, then the *s-extension* of A with k is frequent.

**Property** **3** **(pruning a prefix).** The previous properties can be generalized to prune all patterns starting with a given prefix. Let there be a frequent sequential pattern A and an item k. If there exists an item j∈A (equivalently j in the last itemset of A) such that there is an item $k\in cm_{s}\left(j\right)$ (equivalently in $cm_{i}\left(j\right)$), then all supersequences B having A as prefix and where k succeeds j by *s-extension* (equivalently *i-extension* to the last itemset) in A in B are frequent.

PrefixSpan+ and the original PrefixSpan are based on the same principle to obtain frequent sequential patterns. The difference is that in PrefixSpan+, all the patterns are extended by *i-extension* and *s-extension* of the prefixes, and the completeness is guaranteed by the properties stated above. Then, the whole recursive process is more efficient. 

We refer readers to Reference [27] for more details on CMAP. Now the ideal of PrefixSpan+ and the mining process are illustrated with an example. Given a sequence database (Table 9), suppose minsup=25%, then the threshold of the support number is 2. The aim of the algorithm is to find out all the frequent sequential patterns satisfied with the support requirement.

First, calculate support number of the prefixes with length=1. The results are listed in Table 10. Apparently, (10), (20), (50), and (60) are not frequent sequences under the required minsup, and all the sequences prefixed with them are infrequent, therefore they are deleted from the original database (Table 11). Meanwhile, length-1 sequential patterns are obtained, which are (30), (40), (70), (80), and (90).

Second, construct a CMAP for length-1 sequential patterns from the modified sequence database according to Definition 1–3 (see Table 12). Item j is in the last itemset of a frequent sequential pattern (*i-extension*) or it is a part of a frequent sequential pattern (*s-extension*). In length-1 sequential patterns, j=30,40,70,80,90.

The extensions that are frequent are demonstrated in Property 1–3. We can infer from the properties that if an item k belongs to a certain $cm_{i}\left(j\right)$ or $cm_{s}\left(j\right)$, then the *i-extension*/*s-extension* of the corresponding pattern with k is frequent. That is to say, all the frequent sequential patterns can be obtained through proper extensions. Taking length-1 sequential pattern (30) as example, the length-2 sequential patterns prefixed with (30) can be generated from $cm_{i}\left(30\right)$ and $cm_{s}\left(30\right)$. The results of *i-extension* of (30) with the items in $cm_{i}\left(30\right)$ are (30, 70) and (30, 80), which is the extension in the itemset; the results of the *s-extension* of (30) with the items in $cm_{s}\left(30\right)$ are (30)(40), (30)(70), and (30)(90), which is the extension between the itemsets. In the same way, the length-2 sequential patterns prefixed with (40) and (70) can be obtained, that is, (40, 70) and (70, 80). We can notice that it just needs an *i-extension* since $cm_{s}\left(40\right)=cm_{s}\left(70\right)=\phi$, and there is no need to carry out any extension on (80) and (90), since both of the $cm_{i}\left(j\right)$ and $cm_{s}\left(j\right)$ are empty. Then, all the length-2 sequential patterns are obtained, which are (30, 70), (30, 80), (30)(40), (30)(70), (30)(90), (40, 70), and (70, 80).

Next, by constructing the CMAP for length-2 sequential patterns, it can be found that only structure $CMAP_{i}$ exists on (30)(40) and (30, 70) as shown in Table 13. Therefore, all the length-3 sequential patterns can be obtained through *i-extension*, which are (30)(40, 70) and (30, 70, 80). The CMAP for length-3 sequential patterns are constructed recursively; however, there is no $CMAP_{i}$ or $CMAP_{s}$ that exists, which means no length-4 sequential patterns exist. Then, the mining process is completed, and all the frequent sequential patterns are listed in Table 14.

Through two kinds of extensions, PrefixSpan+ can effectively discover all the frequent sequential patterns without constructing the projection database recursively. The core code of PrefixSpan+ is as shown in Algorithm 1.

**Algorithm 1** The core code of PrefixSpan+**Input:** Sequence dataset S, Threshold of support degree min_sup**Output:** The set of frequent sequential pattern FS1:Scan S, count the support number of all the items with length = 1, and delete the items from S, of which the support degree is less than min_sup, then obtain S’. The set of length-1 sequential pattern P contains all the items that are satisfied with the required support degree.2:FS = P3:
**Extension(S’, P)**
4:**For** each $P_{j}\in P$, construct CMAP for $P_{j}$ from S’5:  **if**
$CMAP_{i}=CMAP_{s}=\phi$
6:    **return** FS 7:  **else**8:    **if**
$CMAP_{i}\neq \phi$, traverse $CMAP_{i}$
9:     extend all the items in $cm_{i}\left(j\right)$ to $P_{j}$ by *i-extension*, and obtain $P_{j}^{i}$
10:    **if**
$CMAP_{s}\neq \phi$, traverse $CMAP_{s}$
11:     extend all the items in $cm_{s}\left(j\right)$ to $P_{j}$ by *s-extension*, and obtain $P_{j}^{s}$
12:    $P_{j}{}^{\prime }=P_{j}^{i}\cup P_{j}^{s}$ // Property 313:    $P^{\prime }=\sum_{j}P_{j}{}^{\prime }$, $FS=FS\cup P^{\prime }$
14:    **Extension(S’, P’)**

### 3.2. Obtain Potential Frequent Sequence

In this section, the sequence-related properties in the updating process are analyzed in depth, then the constitution of the set of potential frequent sequence is confirmed; furthermore, an algorithm named GetPFS is designed to obtain the potential frequent sequences.

**Theorem** **1.** *The new frequent sequences in UD come from the following three parts:*

*1.*  *Frequent sequences in the original SDB;*
*2.*  *The sequences in sdb of which the support number are no less than min_sup×|new|;*
*3.*  *The sequences discovered from the prefix projection database of potential frequent sequence of sdb in DD, and $c^{DD}(\alpha )+c^{new}(\alpha )\geq min\_sup\times \left|new\right|$.*

The proof of theorem 1 is based on the following eight lemmas.

**Lemma** **1.** *The frequent sequences of original database must be contained in the set of potential frequent sequences.*

**Proof.**  To the frequent sequences in original database, the support degree of these sequences in UD cannot be decreased. Owing to $c^{SDB}(\alpha )\geq min\_sup\times \left|SDB\right|$, the value of $c^{SDB}(\alpha )$ is probably more than min_sup×(|SDB|+|new|) such that even the degree of the sequences is not increased in the updated database. Therefore, the potential sequences must contain the frequent sequences in the original database.  □

**Lemma** **2.** *Only the sequences, of which the support number is increased after updating of database, can be the frequent sequences.*

**Proof.**  For the infrequent sequences in the original database, because of $c^{SDB}(\alpha )<min\_sup\times \left|SDB\right|$, $c^{SDB}(\alpha )$ cannot be more than min_sup×(|SDB|+|new|) if the support degree is not increased. □

**Lemma** **3.** *The change of support number will only happen in two kinds of sequences as follows after updating the database.*

*1.*  *The sequences emerged in sdb, including the sequences existing in SDB and the new sequences produced from sdb;*
*2.*  *The new sequences produced from the combination of OD and old.*

**Proof.**  Once the updating of *SDB* is completed, the sequence, of which the support degree has been changed, must be related with *sdb*; that is, either it is from database new or from database old. If the sequence is related with database new, then it is condition 1; if the sequence is related with database old, then it is entirely or partly from old, which fall into condition 1 and 2 respectively. □

**Lemma** **4.** *For each sequence in UD, the inequality $c^{UD}(\alpha )\leq c^{SDB}(\alpha )+c^{sdb}(\alpha )$ holds.*

**Proof.**  For some sequences in UD, the first half part emerges in *SDB*, and the latter part emerges in *sdb*. Besides, some certain sequences may appear in both the *SDB* part and the *sdb* part of a certain sequence. However, the sequences in UD can contribute one to the support number at most, therefore, the inequality $c^{UD}(\alpha )\leq c^{SDB}(\alpha )+c^{sdb}(\alpha )$ holds. □

**Lemma** **5.** *For the sequences in sdb that are infrequent in SDB, the inequation $c^{sdb}(\alpha )\geq min\_sup\times \left|new\right|$ holds if the sequences are frequent in UD.*

**Proof.**  Suppose α is frequent in UD, then $c^{UD}(\alpha )\geq min\_sup\times (\left|SDB\right|+\left|new\right|)$. Therefore:

$$\begin{array}{l} c^{UD}(\alpha )\geq min\_sup\times (\left|SDB\right|+\left|new\right|)\\ \Rightarrow c^{SDB}(\alpha )+c^{sdb}(\alpha )\geq min\_sup\times (\left|SDB\right|+\left|new\right|)\\ \Rightarrow c^{sdb}(\alpha )\geq min\_sup\times \left|SDB\right|-c^{SDB}(\alpha )+min\_sup\times \left|new\right| \end{array}$$

and α is infrequent in *SDB*, then $c^{SDB}(\alpha )<min\_sup\times \left|SDB\right|$, therefore:

$$\begin{array}{l} \Rightarrow c^{sdb}(\alpha )\geq min\_sup\times \left|SDB\right|-c^{SDB}(\alpha )+min\_sup\times \left|new\right|\\ \Rightarrow c^{sdb}(\alpha )\geq min\_sup\times \left|new\right| \end{array}$$

 □

Therefore, if the support number of sequence in *sdb* is no less than min_sup×|new|, the sequence may become frequent; thus, it should be included in the set of potential frequent sequence.

**Lemma** **6.** *If the new sequences produced from the combination of OD and old are frequent, then, for the part from old, it must be included in the set of potential frequent sequences of sdb derived from Lemma 5.*

**Proof.**  If sequence α is frequent, the part from old is also frequent, according to Lemma 5, where the part of α that exists in old is contained in potential frequent sequences. □

**Lemma** **7.** *For the new sequences generated from the combination of OD and old, the support number is*$c^{SDB}(\alpha )-c^{OD}(\alpha )+c^{DD}(\alpha )+c^{new}(\alpha )$.

**Proof.**  There are new sequences that emerge after combination, and the new ones may emerge in new or the original database *SDB*. Owing to the support number of a same sequence, from DD and OD, it may calculate twice or even more times, for instance, in the case of $<ad\underline{ad}>$; thus, the contribution from OD should be subtracted from the sum of the support number in *SDB*, DD, and new during the calculation. □

**Definition** **4.** *Suppose sequences α,β,λ, with β⊆α,λ⊆α. λ is a postfix projection of α to β if the following requirements are satisfied: (1) β is the postfix of λ; (2) there does not exist a sequence $\lambda^{\prime }$ that satisfies $\lambda \neq \lambda^{\prime },\lambda^{\prime }\subseteq \alpha ,\lambda \subseteq \lambda^{\prime }$, and β is the postfix of $\lambda^{\prime }$.*

The attribute of postfix projection is described in Definition 4, which means a postfix projection λ to β is the maximum subsequence of α postfixed with β. For instance, given a sequence α=<a(abc)(ac)d(cf)>, if β=<(cf)>, then the postfix projection λ=<a(abc)(ac)d(cf)>; if β=<(ac)>, then λ=<a(abc)(ac)>.

**Definition** **5.** *Suppose there exist sequences $\alpha =<X_{1}X_{2}\ldots X_{i}\ldots X_{n}>,\beta =<{X^{\prime }}_{i}X_{i+1}\ldots X_{j}>$, with $X_{i}={X^{\prime }}_{i}+{X^{\prime \prime }}_{i}$1≤i≤j≤n, then the postfix projection λ is $<X_{1}X_{2}\ldots X_{i}\ldots X_{j}>$ according to Definition 4. Sequence $\gamma =<X_{1}X_{2}\ldots {X^{\prime \prime }}_{i}>$ which is the rest of λ except $<{X^{\prime }}_{i}X_{i+1}\ldots X_{j}>$, and is called a prefix of α to β, and denoted as γ=λ/β. Let SDB be a sequence database, and α is a sequence in SDB, then the set of prefixes from projections of all the sequences in SDB to β, is called a prefix database of the projection of *SDB* to β, and denoted as ${suffix\text{-}SDB\;|}_{\beta }$.*

For instance, there are two sequences in *SDB*, which are $\alpha_{1}=<a(abc)(ac)d(cf)>$ and $\alpha_{2}=<(bd)(cf)>$, and β=<(cf)>. For $\alpha_{1}$, $\lambda_{1}=<a(abc)(ac)d(cf)>$ and $\gamma_{1}=<a(abc)(ac)d>$; for $\alpha_{2}$, $\lambda_{2}=<(bd)(cf)>$ and $\gamma_{2}=<(bd)>$. Then, the ${suffix\text{-}SDB\;|}_{\beta }$ is {<a(abc)(ac)d>,<(bd)>}.

Based on the ideal of PrefixSpan+, a “PostfixSpan+” algorithm can be obtained through reversing the direction of *i-extension* and *s-extension* during the extending process; for instance, for a sequence <(30)(40,70)>, the *i-extension* of item 40 is 70, and the *s-extension* is ϕ in PrefixSpan+. In PostfixSpan+, the *i-extension* of item 40 is ϕ, and the *s-extension* is 30. In the following, the Postfix algorithm is utilized to process the new sequences produced from the combination of OD and old.

**Lemma** **8.** *Construct suffix-SDB to the potential frequent sequences of sdb in DD. If the new sequences produced from the combination of OD and old are frequent, then they must be included in the sequences generated from the suffix-SDB*; also, the inequation*$c^{DD}(\alpha )+c^{new}(\alpha )\geq min\_sup\times \left|new\right|$ holds.*

**Explanation.**  All the sequences from old are already involved in potential frequent sequences of *sdb*, therefore they do not need to be the elements of the prefix projection database. For instance, sequence $<ab\underline{aa}>$ and potential frequent sequence a; then, the prefix of projection is not $<ab\underline{a}>$, but <ab>.

**Proof.**  The sequences generated from the combination of OD and old are frequent, thus the part of them, which are from *sdb*, must be potential sequences; meanwhile, to the prefix projection database constructed by all the potential frequent sequences, the frequent sequences generated from the combination of OD and old are involved in it. Then, the following inequation holds:

$$\begin{array}{l} c^{UD}(\alpha )\geq min\_sup\times (\left|SDB\right|+\left|new\right|)\\ \Rightarrow c^{SDB}(\alpha )-c^{OD}(\alpha )+c^{DD}(\alpha )+c^{new}(\alpha )\geq min\_sup\times (\left|SDB\right|+\left|new\right|)\\ \Rightarrow c^{DD}(\alpha )+c^{new}(\alpha )\geq min\_sup\times \left|SDB\right|-c^{SDB}(\alpha )+c^{OD}(\alpha )+min\_sup\times \left|new\right|\\ \Rightarrow c^{DD}(\alpha )+c^{new}(\alpha )\geq c^{OD}(\alpha )+min\_sup\times \left|new\right|\\ \Rightarrow c^{DD}(\alpha )+c^{new}(\alpha )\geq min\_sup\times \left|new\right| \end{array}$$

 □

Therefore, if the sum of the support numbers, in DD and new, of the sequences produced by the combination OD and old is no less than min_sup×|new|, they might become frequent sequences, which means they should be involved in the set of potential frequent sequence.

Therefore, we conclude from the above lemmas that if a sequence is frequent in UD, then it must be satisfied by one of the conditions mentioned above, which means it will be added into the set of potential frequent sequence. It just needs two steps to obtain a potential frequent sequence:

Step one: Implement sequence mining on *sdb* by using PrefixSpan+, setting the threshold of the support number as min_sup×|new|;

Step two: Construct the prefix projection database under DD to potential frequent sequences in *sdb*, then implement sequence mining on the constructed databases by using PostfixSpan+, setting the threshold of the support number as min_sup×|new|.

Suppose the FS (frequent sequence) is the set of frequent sequences in *SDB*, PFS (potential frequent sequence) as the set of potential frequent sequence, and NFS (new frequent sequence) is the set of frequent sequence in updated database. The core code of algorithm GetPFS, which is used to obtain the potential frequent sequences, is described in Algorithm 2:

**Algorithm 2** GetPFS (OD, DD, FS, old, new)**Input:** OD, DD, FS, old, new**Output:** PFS1:**Put** FS **into** PFS2:Set s1=PrefixSpan+({},old+new,min_sup×|new|)
3:**Put**s1**into** PFS4:**For**b**in** PFS5:  Construct projection database S of DD to the part of b that belongs to OD6:  Set s2=PostfixSpan+(b,S,min_sup×|new|)
7:  **Put**
s2
**into** PFS

### 3.3. Fast Support Number-Counting Algorithm

**Figure 2:**
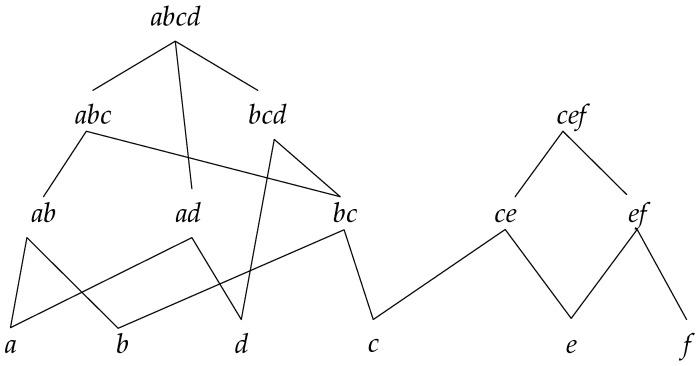
Reticular Sequence Tree.

In order to calculate the support degree once the potential sets of frequent sequence (PFS) are obtained, a fast counting algorithm is proposed in this section. To level up the efficiency of calculating, a reticular structure is designed to store the PFS, making the speed of counting faster than the traditional methods such as a hash function-based algorithm. Figure 2 shows a reticular sequence tree structure, with PFS={abcd,abc,bcd,cef,ab,ad,bc,ce,ef,a,b,d,c,e,f}.

In this tree structure, abcd and cef are the root nodes, and each child node is the subsequence of the parent node; also, if a sequence is the subsequence of another, then it must be in the reticular sequence tree with the parent sequence as root node.

**Figure 3:**
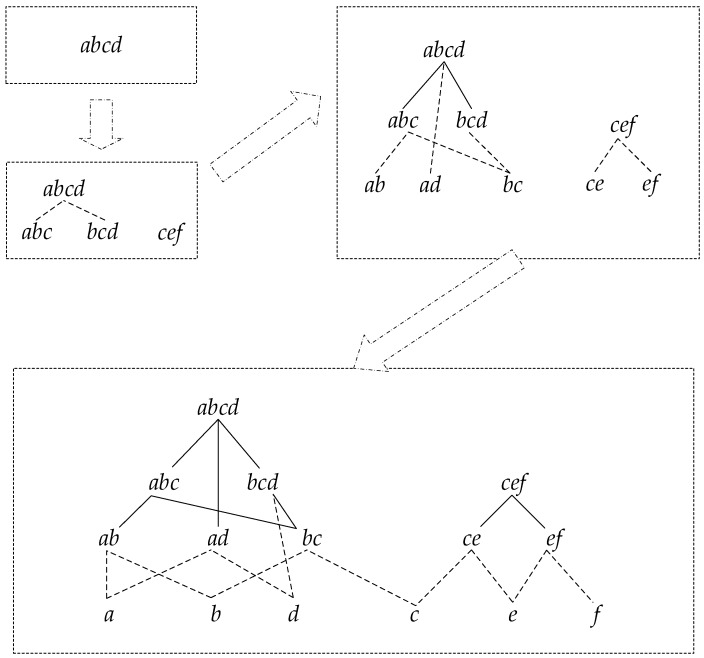
The Construction Process.

The concrete process for constructing a reticular sequence tree is as follows. Each layer is comprised of the sequences with the same length. The first layer is the set of the longest sequences, and each node is a root node. Then, check the rest of the sequences in decreasing order to determine if they are the subsequence of the node in the higher layer; if so, add them as the corresponding child node, and if not, set them as the root node. Figure 3 shows the detailed construction process.

**Figure 4:**
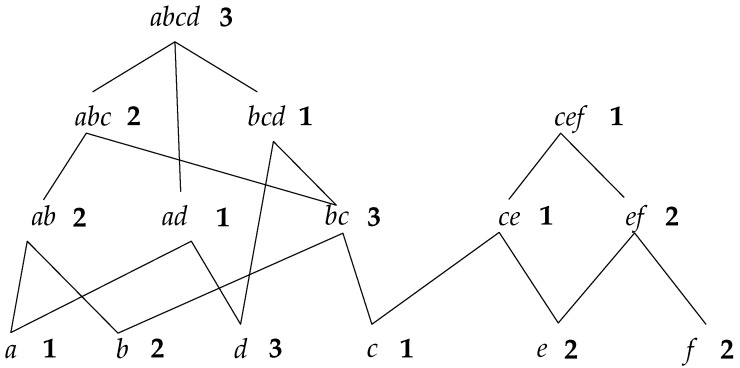
The Counted Results.

The support number of each node can be obtained through two steps. First, count the elementary support number. Check whether the sequence contains a root node traversing all the sequences in the updated database; if so, the support number of the root node adds one. If not, check if it contains the sequence of the child node; if so, the support number of the child node adds one, and if not, check recursively. For instance, if there is a sequence <(abcde)> in the database, for root node abcd, which is contained in the sequence, then its support number adds one; if the sequence is <(abc)>, it does not contain root node abcd, but for its child node abc, the support number should add one. We can notice that the child nodes do not need to be checked if there is any father node included in the sequence. Supposing there is a set of results counted from step one, as shown in Figure 4, the number on the right side of each node is the elementary support number.

**Figure 5:**
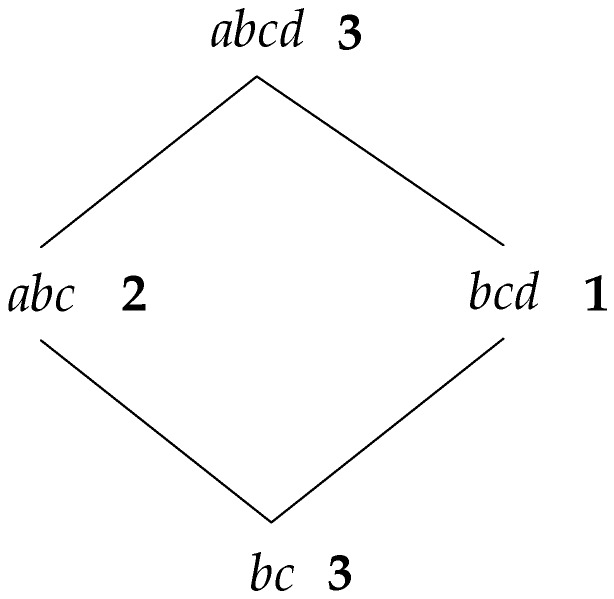
Diamond Structure.

In step two, the real support number of each node is obtained by calculating the sum of elementary support number of itself and all the father nodes. For instance, as shown in Figure 4, the elementary support number of node ab is 2, and the numbers of its father nodes abc and abcd are 2 and 3, respectively; therefore, the real support number of ab is 7. However, for node bc, it has three father nodes—abc,bcd, and abcd—where abcd is the common father node of abc and bcd; then, its elementary support number can just be counted once, so the real support number of bc is 9. That is to say, in a diamond structure like Figure 5, the elementary support number of all the common father nodes can only be added one time when counting the real support number.

In the real programming process, counting the real support number and determining whether the sequence is frequent or not could be carried out simultaneously. First, the support number is calculated from a leaf node: if it is equal or greater than the minimum support number, then determine whether its father node is frequent; if the support number is less than the minimum support number, it can be confirmed that all the father nodes are not in the frequent sequence.

To sum up, the fast support number-counting algorithm is comprised of three steps:

1. Construct a reticular sequence tree;
2. Count the support number on the constructed reticular sequence tree by traversing the updated sequence database;
3. Count the real support number and determine the frequent sequences.

## 4. Experimental Analysis

**Figure 6:**
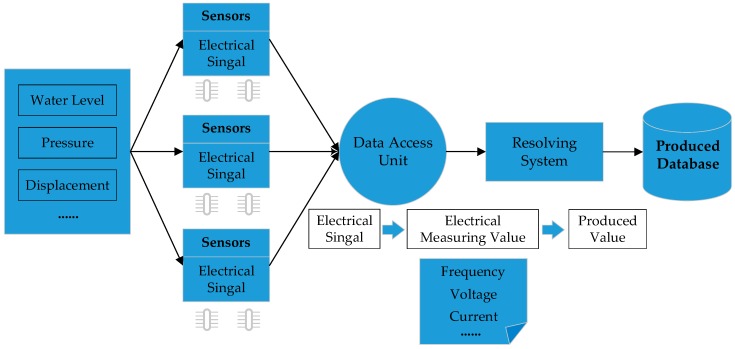
The Monitoring Data Flow.

The experiments were carried out on the following configurations, using Java (jdk 1.7): CPU (Dual-Core CPU E5300 2.60 GHZ); Memory (4 GB); 64-bit Windows 8. The experiments are conducted on two benchmarking datasets from real-life (BMSWebView-1 and Kosarak) [28], two synthetic datasets [28], and real monitoring datasets. The monitoring data, that is, horizontal displacement of a dam and water level of a reservoir, was collected from a huge hydropower plant in China. The horizontal displacement was detected by a specific kind of capacitive sensor embedded in the dam. It reported the capacitance ratio to a DAU (data access unit) periodically, and the horizontal displacement could be obtained using a piecewise function based on the ratio. The water level was measured using a water level sensor. It transformed the perceived signals into electrical signals in real time, and forwards them to the DAU, then the value of water level can be calculated. More specifically, the original electrical signals were first transmitted to the DAU and then the electrical measuring values (frequency, voltage, or current) were calculated by MCU (microcontroller unit) based on the relevant model. Finally, by using the measuring values, the results that represent the corresponding states, called produced value, could be obtained through resolving system. The data flow is illustrated in Figure 6.

A sequence was comprised of the value of horizontal displacement and water level, as well as other related information, such as time, position, and pressure. The original sequence database *SDB* was constructed by selecting appropriate amounts of historical monitoring data from “Produced Database,” which is an SQL server in practical use. The correlation between horizontal displacement and water level can be discovered through mining sequential patterns on *SDB*. The sequences were formatted in accordance with the data generated in a standard procedure [17]. The experimental parameters are listed in Table 15.

### 4.1. Generation of Incremental Database

The updated database UD is comprised of original database *SDB* and incremental database *sdb*, which can be created in the following way. First, set the corresponding parameters |UD|, $R_{sdb}$, and $R_{new}$. $R_{sdb}$ is the ratio of incremental database *sdb* to the updated database UD; that is to say, it defines the size of *sdb*: $\left|sdb\right|=\left|UD\right|\times R_{sdb}$. Generate |sdb| sequences from historical monitoring data in a standard format to form *sdb*, and *SDB* is constructed in the same way. $R_{new}$ is the ratio of the new sequences to the incremental database *sdb*; then, randomly select $\left|sdb\right|\times R_{new}$ sequences from *sdb* as new sequences, and split the rest of sequences into two parts, for instance, $<X_{1}X_{2}\ldots X_{l}>$ is split into $<X_{1}X_{2}\ldots X_{m}>$ and $<X_{m+1}X_{m+2}\ldots X_{l}>$. Finally, the first parts of all the split sequences are added into *sdb*, and the latter parts are inserted into *SDB*.

### 4.2. Comparisons

We conducted substantial experiments on the real monitoring datasets in different sizes, two benchmarking datasets and two synthetic datasets, to compare the time cost, the sensitivity of $R_{sdb}$ and $R_{new}$, and the space cost with various kinds of existing approaches.

#### 4.2.1. Time Cost on Real Monitoring Datasets

The time cost was first compared with some typical methods on monitoring datasets, including PrefixSpan [17], PFT (Pre-large FUSP-Tree) [20], IncSpan [29], and STISPM (Sequence Tree-based Incremental Sequential Pattern Mining) [30]. The parameters were set to be C=10,T=4, S=12,I=4, and the number of different elements N=100K,10K, and $R_{sdb}=5\%,R_{new}=50\%$. The experiment was carried out on two sets of data (N=100K,N=10K) with different support degrees (minsup=3%,2%,1%, and 0.75%).

**Figure 7:**
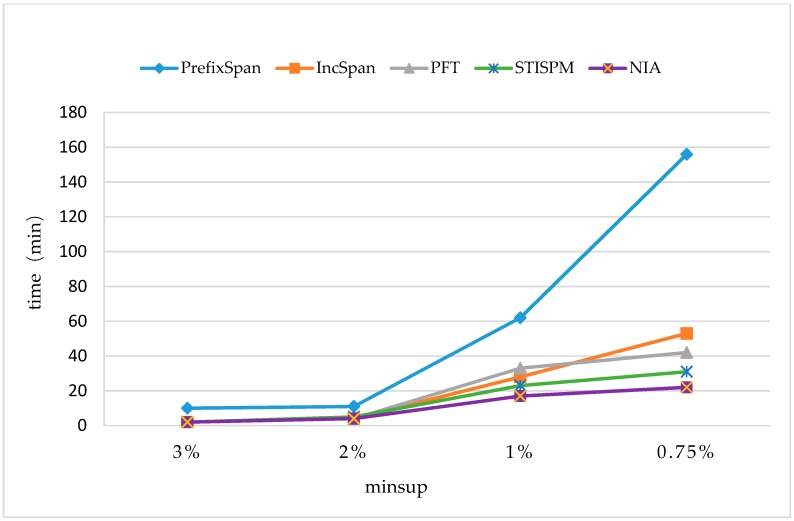
The Runtimes on the Dataset of |UD|=100K with N=100K.

**Figure 8:**
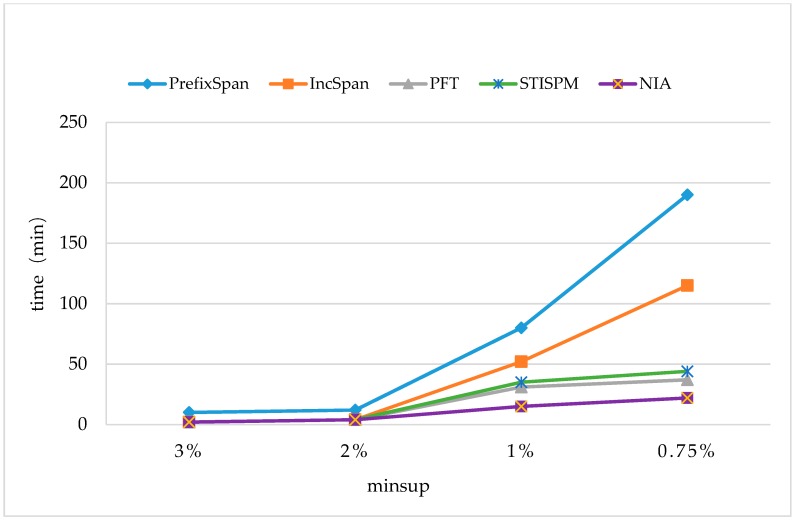
The Runtimes on the Dataset of |UD|=100K with N=10K.

In Figure 7 and Figure 8, it shows that all the incremental mining algorithms, Incspan, PFT, STISPM, and the proposed NIA, performed better on efficiency than PrefixSpan, which needed to re-mine the whole database after updating. Furthermore, NIA, which reduced the time cost by 85.9% and 88.4% at most, compared to PrefixSpan, had the best performance on efficiency for all incremental algorithms generally.

In Figure 7, NIA had a lower time cost than Incspan, PFT, and STISPM as minsup=2%,1%, and 0.75%; on average, it reduced the time cost by 49.4% compared to Incspan, 45.6% compared to PFT, and 27.1% compared to STISPM. In Figure 8, NIA still performed the best in all the incremental algorithms; more specific, there were 75.1%, 41.9%, and 49.4% decreases on average compared to Incspan, PFT, and STISPM, respectively. Furthermore, the growth rate of NIA’s time cost was still the lowest during minsup changes from 2% to 1%, both in Figure 7 and Figure 8, but in the process of 1% to 0.75%, although NIA is more efficient, its growth rate was slightly faster than PFT. This was owed to the amount of potential frequent sequences obtained by NIA that was greatly increased with the decrease of minsup, resulting in a heavier computation burden on constructing the reticular sequence tree and counting the support number. Nevertheless, the minsup was commonly not too small in real applications to avoid obtaining so many useless frequent sequences.

Through the comparison between Figure 7 and Figure 8, it could be found that the runtimes of all the algorithms were reduced when N=100K. The reason was the amount of frequent sequences being cut down with the augment of different elements in the database, making the execution process faster. For instance, NIA discovered 313 frequent sequences in one hundred thousand sequences when N=100K,minsup=1%, but there were 714 sequences in one hundred thousand sequences when N=10K,minsup=1%. It also indicates that NIA was more sensitive to the amount of frequent sequences than other three algorithms. That is why NIA performed more efficiently when N=100K.

In order to further evaluate the performance of the proposed scheme, we enlarged the scale of UD to 200K, and tested the time cost with the settings of $C=10,T=4,S=12,I=4,R_{sdb}=5\%,R_{new}=50\%$, and N=10K.

**Figure 9:**
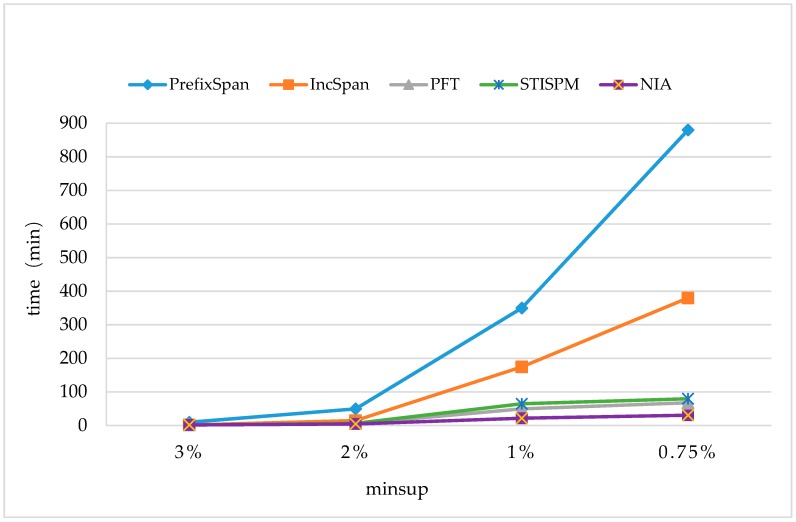
The Runtimes on the Dataset of |UD|=200K with N=10K.

From Figure 9, we found that the runtime of original PrefixSpan was intolerable as the mining space doubled, and the time consumption of NIA was 15.0875 mins on average, which grew sub-linearly with the augment of the amount of sequences in UD; also, compared to the runtimes of IncSpan, PFT, and STISPM, which were respectively 143.1125, 31.625, and 38.6875 mins on average, NIA reduced the time cost by 89.5%, 52.3%, and 61%.

**Figure 10:**
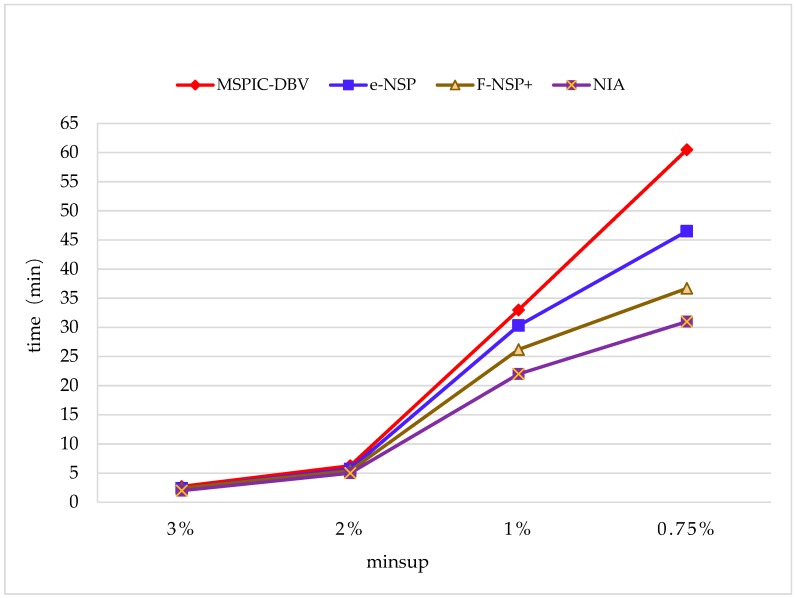
The Runtime Comparison with Three Novel SPM Algorithms.

In addition, there were several up to date SPM algorithms that achieved favorable performance based on novel ideals and good design, and three of them, MSPIC-DBV (Mining Sequential Patterns with the Itemset Constraints using Dynamic Bit Vector) [31], e-NSP (Efficient Negative Sequential Pattern mining) [32], and F-NSP^+^ (Fast Negative Sequential Patterns mining) [33], were selected for further comparisons (See Figure 10) on the monitoring dataset. The parameter settings were $C=10,T=4,S=12,I=4,\left|UD\right|=200K,N=10K,R_{sdb}=5\%,R_{new}=50\%$.

MSPIC-DBV uses a dynamic bit vector to represent the sequences containing the candidate patterns and proposes a tree structure named DBVS (Dynamic Bit Vector for Sequential patterns) prefix-tree for mining sequential patterns efficiently; however, it needs to rescan the whole database for updating the discovered patterns. It can be found that MSPIC- DBV performed the worst in the comparison, and the time cost was 25.6125 mins on average, which was 70.75% more than NIA. e-NSP is an efficient SPM algorithm that does not rely on database rescans, and F-NSP^+^ is an improved version of e-NSP. e-NSP uses an array to store the information of sequential patterns and uses a hash method to obtain the support. e-NSP is good at handling sparse datasets, but it is inefficient when datasets become dense, owing to the key process requiring a much greater time consumption. Therefore, in Figure 10, it can be seen that the runtime of e-NSP grew faster when minsup was decreased. The average time cost of e-NSP was 21.225 mins, which was 41.5% more than NIA. F-NSP^+^ uses a novel data structure named bitmap to store the information of sequential patterns, and the support can be obtained using bitwise operations, and although it is much faster than the hash method used in e-NSP, the support number-counting algorithm designed in NIA is more efficient. The average time cost of F-NSP^+^ was 17.675 mins, which was 17.8% more than NIA.

**Figure 11:**
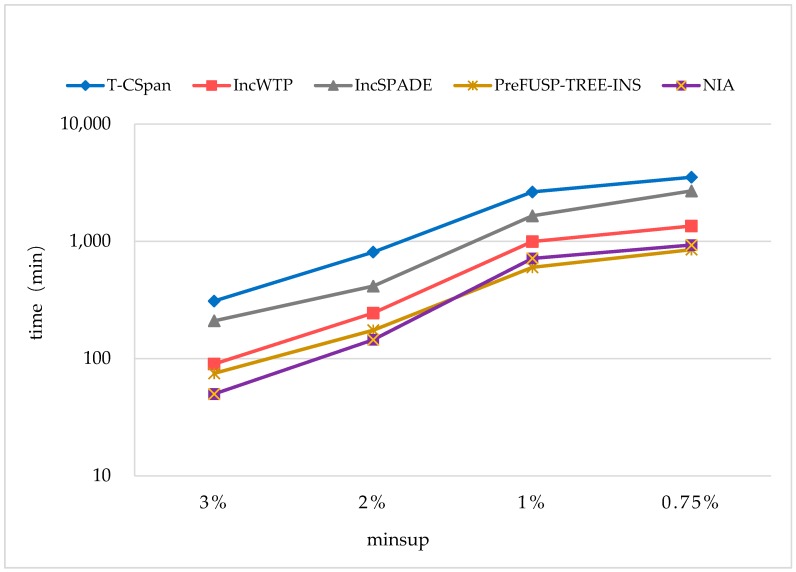
The Runtime Comparison with the Practical SPM Algorithms.

Furthermore, the historical monitoring data for two years was collected to construct a large-scale dataset with |UD|=10000K for testing the NIA’s performance under a big data environment; that is, there are 10 million sequences in the searching area. The comparing objects included the practical algorithms, T-CSpan [34], IncWTP [35], IncSPADE [36], and PreFUSP-TREE-INS [21] (see Figure 11). The parameter settings were C=10,T=4,S=12,I=4,|UD|=10000K,N=10K and $R_{sdb}=5\%,R_{new}=50\%$.

T-CSpan was proposed for finding optimal clinical pathways by using an improvement of PrefixSpan. It utilizes an occurrence check to reduce the mining space; however, the checking procedure needs to be executed repeatedly once the database is updated. From Figure 11, it can be seen that T-CSpan performed the worst of all the compared algorithms, and the runtime was over $3.5\times 10^{3}$ mins when minsup=0.75%. In NIA, the mining space was greatly narrowed based on the proof of completeness without any checking or other procedures. IncSPADE is a tree structure-based algorithm, and the information of frequent/infrequent sequences is saved in a tree map; then, dynamic databases can be handled incrementally through maintaining the tree structure. However, the algorithm is not succinct, which contains many checking and determining processes. Furthermore, the phase of scanning the updated database depends on the size of the database, which may lead to high computation complexity. Therefore, we found that its performance was unfavorable, and the time cost was over $1.5\times 10^{3}$ mins when minsup≤1%. IncWTP also uses a grid structure to save previous mining outcomes attached with supported counting information, and different from NIA, it updates the grid structure with deletion and insertion when the corresponding changes happened in the database, and the previously found information is utilized to discover updated sequence access mode, without re-excavation of the entire database. However, IncWTP is designed for a web log database, and it does not achieve preferable performance in the constructed database.

PreFUSP-TREE-INS is a recognized algorithm in incremental mining, which achieves better time performance than other well-known algorithms with a proper space cost. In the mining process, the database is scanned only when the cumulative number of newly added customer sequences exceeds a safety bound by using the pre-large concept; then, the number of database rescans is reduced greatly. Particularly, the PreFUSP-tree structure, which is proposed for efficiently maintaining discovered large sequences, can be easily updated without a re-mining process when the database is changed. We found that the average time cost of PreFUSP-TREE-INS was close to that of NIA, and PreFUSP-TREE-INS performed slightly better than NIA when minsup≤1%.

#### 4.2.2. Time Cost on Benchmarking Datasets and Synthetic Datasets

**Figure 12:**
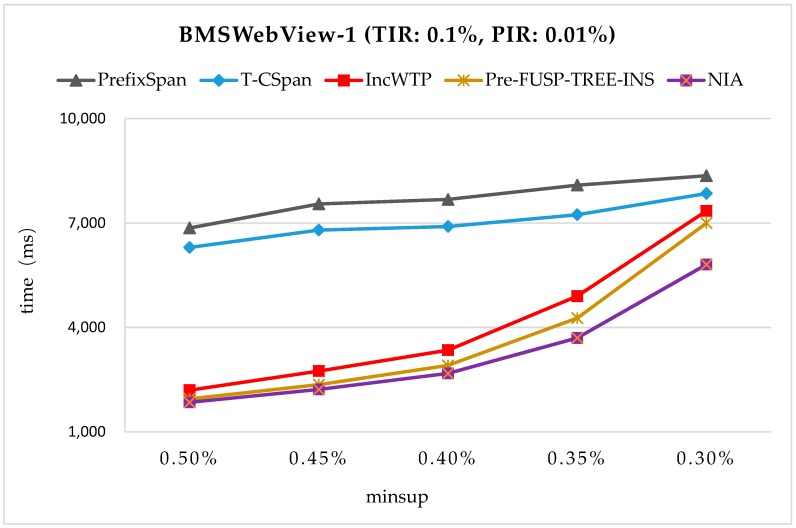
The Runtimes on BMSWebView-1 with TIR = 0.1% and PIR = 0.01%.

**Figure 13:**
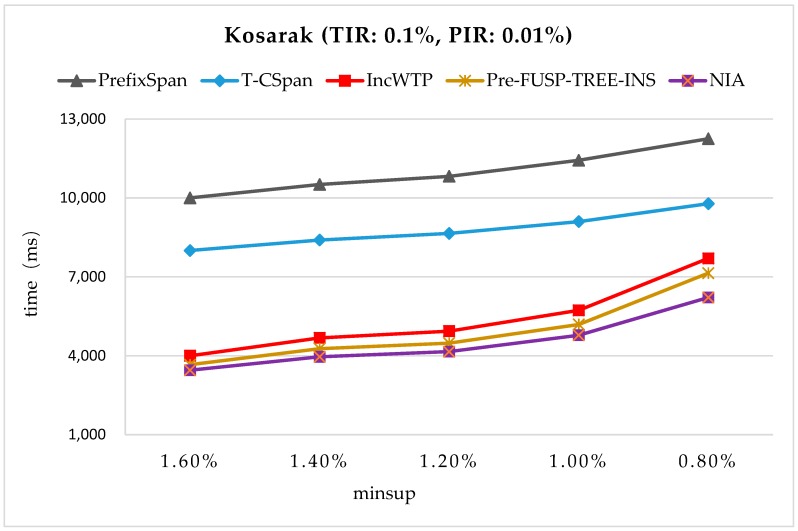
The Runtimes on Kosarak with TIR = 0.1% and PIR = 0.01%.

The time efficiency was also tested on the datasets that are commonly used in the pattern mining field. First, we ran NIA on two benchmarking datasets BMSWebView-1 [28] and Kosarak [28], and the time costs were compared between PrefixSpan [17], T-CSpan [34], IncWTP [35], and PreFUSP-TREE-INS [21] (see Figure 12 and Figure 13). BMSWebView-1 contained several months of click-stream sequences from an e-commerce website. There were 59,601 sequential transactions and 497 distinct items. The average number of distinct items was 2.51 and the maximum size of transactions was 267. Kosarak is a public database that contained 990,000 click-stream sequences from the logs of an online news portal. Only the first 70,000 sequences were used in the experiments, with 21,144 distinct items and 7.97 items on average. In the experiments, the total insertion ratio (TIR) was fixed and the minimum support thresholds were varied. For BMSWebView-1, the support threshold was changed from 0.3% to 0.5%. The TIR was set at 0.1% of the original database (=60 sequences), and the partial insertion ratio (PIR) was set at 1/10 of the TIR (=6 sequences). For Kosarak, the minimum support threshold was changed from 0.8% to 1.6%. The TIR was set at 0.1% of the original database (=70 sequences), and the PIR was set at 1/10 of the TIR (=7 sequences).

From Figure 12 and Figure 13, it can be observed that NIA performed the best in the comparison since it reduced the mining space and improves the mining algorithm. PrefixSpan and T-CSpan have longer runtimes than the others since they both needed some repeated operations to mine the updated sequential patterns. IncWTP and PreFUSP-TREE-INS utilized different structures to maintain the discovered knowledge in order to enhance the mining efficiency, and they all perform relatively well in the benchmarking test.

**Figure 14:**
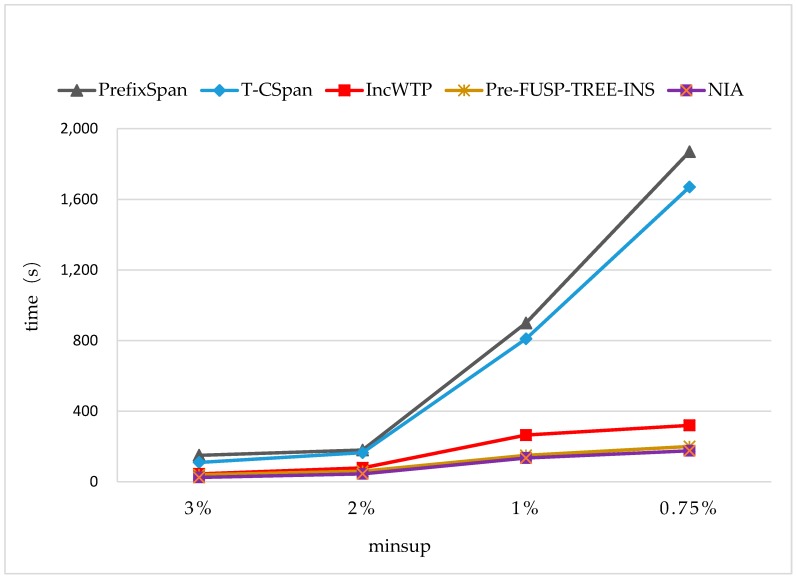
The Runtimes on the Synthetic Dataset 1 with $R_{sdb}=5\%,R_{new}=50\%$.

**Figure 15:**
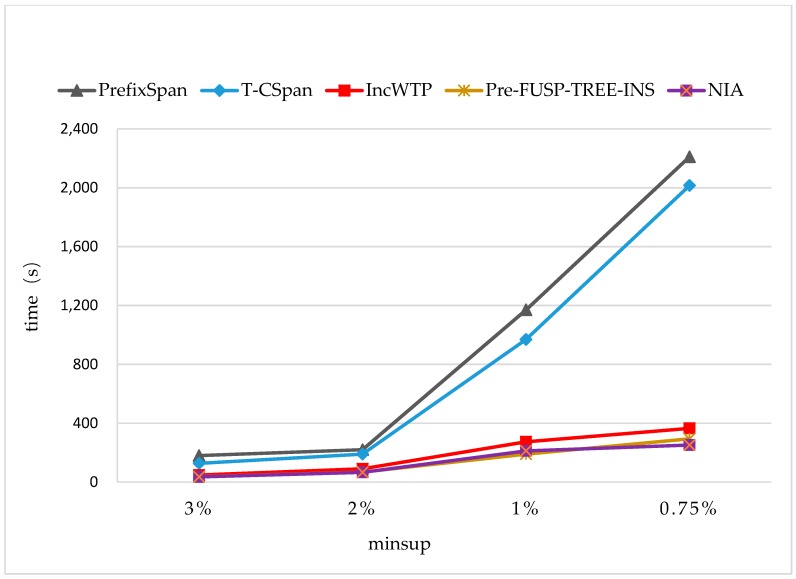
The Runtimes on the Synthetic Dataset 2 with $R_{sdb}=5\%,R_{new}=50\%$.

The time costs were also evaluated on two synthetic datasets [37,38] (see Figure 14 and Figure 15). The average sequence length slen was 10 and the average length of pattern seq.patlen was 2 in the synthetic dataset 1, which contains 50,350 sequences. The slen was 8 and seq.patlen was 4 in synthetic dataset 2, which contained 46,130 sequences. We also used the way specified in Section 4.1 to generate the incremental database, and set $R_{sdb}=5\%,R_{new}=50\%$. In the experiments, the minimum support threshold was changed from 3% to 0.75%.

From Figure 14 and Figure 15, it can be seen that NIA still performed better than the other algorithms, but the average time cost was increased by 46 seconds compared to the results on synthetic dataset 1. The reason lies in the average sequence length and the average length of pattern that were all changed in the new dataset, making the times of the extensions (*i-extension* and *s-extension*) implemented during the mining process of NIA longer. IncWTP and PreFUSP-TREE-INS also performed better than PrefixSpan and T-CSpan. Especially, PreFUSP-TREE-INS was suitable for synthetic dataset 2. The average time cost was just 6 s more than that of NIA, and it cost less time than NIA when minsup=1%. 

In the following, the sensitivity of $R_{sdb},R_{new}$, and the space cost were evaluated on the real monitoring data.

#### 4.2.3. Sensitivity of $R_{sdb}$ and $R_{new}$

In order to observe the sensitivity of $R_{sdb}$ and $R_{new}$, the time performances of NIA and other compared methods, including IncSpan, PFT, and STISPM, were tested with the changing of these two factors.

**Figure 16:**
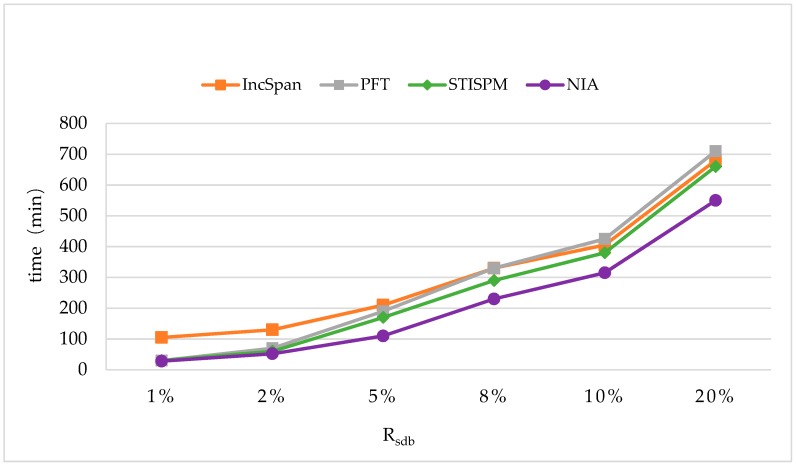
The Runtimes with the Different Settings of $R_{sdb}$.

Figure 16 shows the variation of runtimes as $R_{sdb}$ changes from 1% to 20%. The parameter settings were C=10,T=4,S=12,I=4,|UD|=100K,N=100K, and $R_{new}=50\%,\min\sup=0.75\%$.

The runtimes of four algorithms all rose, along with the growing of $R_{sdb}$, and the proposed algorithm NIA performed better than the other three algorithms; compared to them (IncSpan, PFT, and STISPM), the time cost was reduced by 30.9%, 26.8%, and 19.2%. For the growth rate of the time cost, NIA was not faster than the other algorithms generally when $R_{sdb}$ changed from 1% to 20%; it was even sensitive to the amount of frequent sequences. This is because the efficiency of mining process on *sdb* was enhanced by utilizing the designed PrefixSpan+, making the whole runtime reduced.

**Figure 17:**
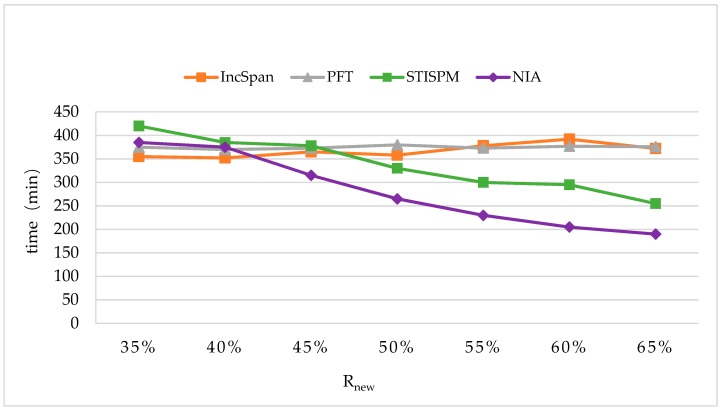
The Runtimes with the Different Settings of $R_{new}$.

Figure 17 shows the variation of runtimes as $R_{new}$ changes from 35% to 65%. The parameter settings were C=10,T=4,S=12,I=4,|UD|=100K,N=100K, and $R_{sdb}=10\%,\min\sup=2\%$.

It is apparent that NIA’s runtime was longer than IncSpan and PFT when $R_{new}<40\%$; however, its efficiency was sharply enhanced since $R_{new}>40\%$, so $R_{new}$ brought a greater effect on the proposed NIA. This was because the setting of the threshold value during obtaining the potential frequent sequences heavily relied on $R_{new}$. If $R_{new}$ was too small, a large number of potential frequent sequences will be obtained, resulting in a heavy computation burden on constructing the reticular tree and counting support number; correspondingly, when $R_{new}$ became larger, massive infrequent sequences were filtered during discovering frequent sequences, therefore the efficiency was lifted up quickly. STISPM performed the worst in all algorithms when $R_{new}<45\%$, owing to having to consider so many infrequent and irregular patterns produced by a large number of potential frequent sequences. When $R_{new}>45\%$, it performed more efficiently, but NIA had the advantage on mining potential frequent sequences and counting the support number by suggesting corresponding methods; thus, NIA had the best performance since $R_{new}>40\%$.

Extracting frequent sequences from the set of potential frequent sequences consumed most of the computation cost, so the amount of potential frequent sequences was closely related to the performance of the mining algorithms.

**Figure 18:**
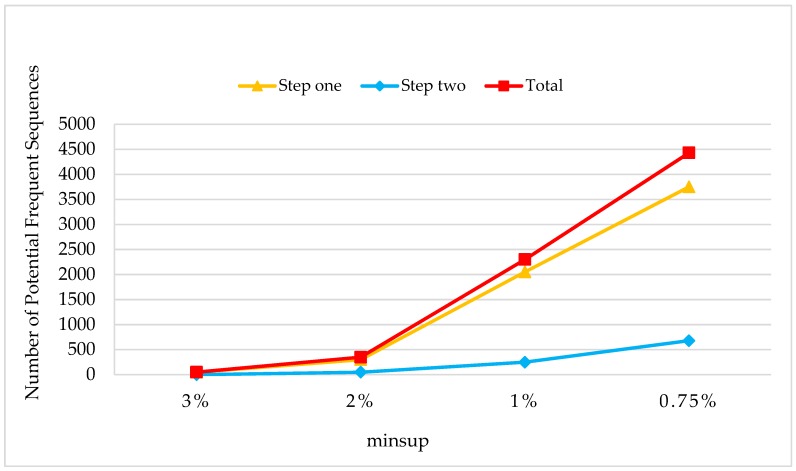
The Amounts of Potential Frequent Sequences on the Different minsups.

Figure 18 shows the amounts of potential frequent sequences obtained in a two-steps phase (Section 3.2.) with the change of minsup. The parameter settings were C=10,T=4,S=12,I=4,|UD|=100K,N=100K, and $R_{sdb}=10\%,R_{new}=50\%$. The numbers of potential frequent sequences were increased linearly in each step with the minsup changes from 3% to 0.75%, and more sequences were obtained in step one with a higher growth rate.

#### 4.2.4. Space Cost

**Figure 19:**
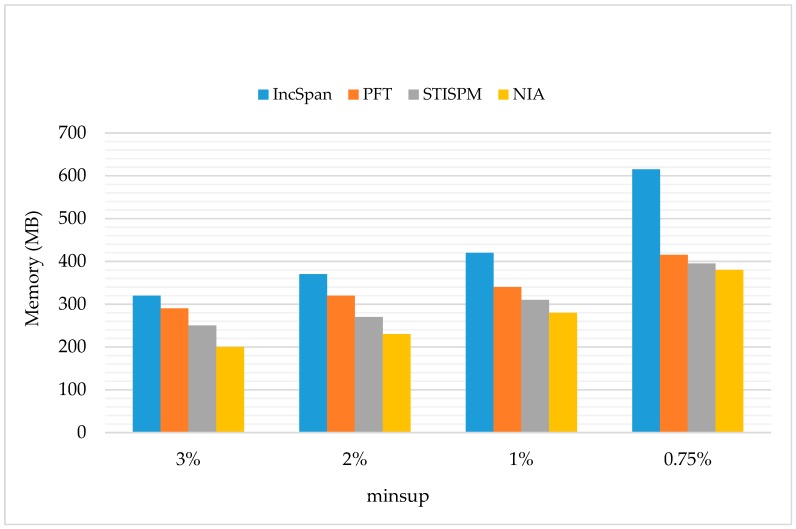
The Memory Usage on the Dataset of |UD|=100K with N=100K.

**Figure 20:**
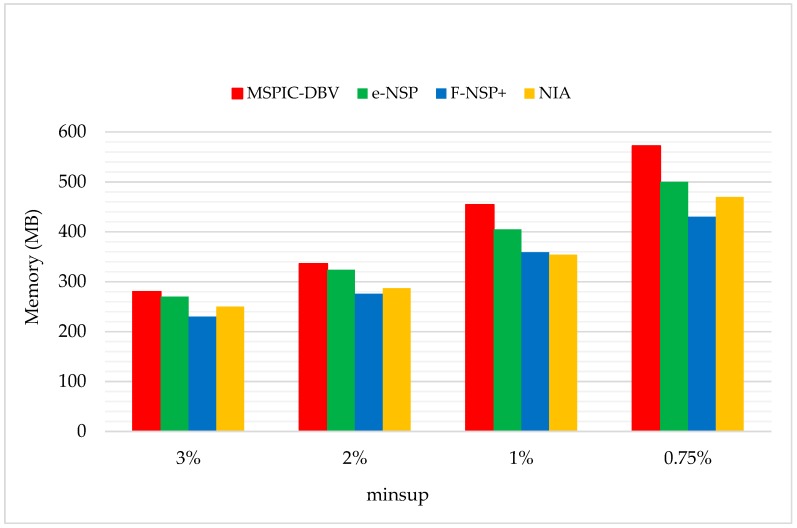
The Memory Usage Comparison with Three Novel SPM Algorithms.

The space cost of NIA was evaluated on real monitoring datasets, and the results were compared with three incremental algorithms (IncSpan, PFT, and STISPM; see Figure 19), and three novel SPM algorithms (MSPIC-DBV, e-NSP, and F-NSP^+^; see Figure 20).

Figure 19 shows the memory consumption of four incremental algorithms. The parameter settings were C=10,T=4,S=12,I=4,|UD|=100K,N=100K, and $R_{sdb}=10\%,\;R_{new}=50\%$. NIA used less memory than all the other algorithms. This was because in the process of sequence mining, the execution of NIA was just put on part of sequences, such that there was no need to pass the original database *SDB* and the incremental database *sdb* into memory at the same time. Furthermore, NIA is based on PrefixSpan+, which has a steady memory consumption of storing the structure CMAP and the obtained potential frequent sequences.

Regarding STISPM, it needs to access the set of sequences frequently in order to realize precise pruning, and a sequence tree needs to be stored during the execution process; thus, it has a greater memory use than NIA. However, when the minsup is too small, massive obtained potential frequent sequences need to be stored in the memory, making the growth rate of NIA’s memory cost faster than PFT and STISPM.

Figure 20 shows the memory usage comparison between NIA and three novel SPM algorithms. The parameter settings were C=10,T=4,S=12,I=4,|UD|=200K,N=10K, and $R_{sdb}=10\%,R_{new}=50\%$. MSPIC-DBV uses a prefix-tree structure to arrange the candidate patterns, which is similar to NIA, but some key information about the original database must be pre-stored into the memory to carry out the pruning process once the dataset is updated. Therefore, it can be found that MSPIC-DBV performed the worst in the comparison. e-NSP stores all the IDs of sequences in the patterns in an array, and the number of sequence IDs was large, even when the minsup was high. Thus, when the minsup was reduced, a large number of sequential patterns were generated, making e-NSP consume larger amounts of storage space. F-NSP^+^ is incorporated with a self-adaptive storage strategy, which automatically chooses a bitmap or an array to store the sequential patterns’ information according to the minsup, and the space efficiency is improved compared to e-NSP. Also, it performed slightly better than NIA in general, but not steadily. For instance, it cost 276.35 MB memory space when minsup=2%, which was less than the 287.64 MB cost by NIA; however, when minsup=1%, F-NSP^+^ cost 359.72 MB memory space, which was more than the 354.23 MB cost of NIA, and when minsup changed to 0.75%, the space cost was less than NIA again. This was because the storage strategy of F-NSP^+^ is chosen in accordance with the varying minsup.

## 5. Conclusions

Based on the analysis of the current research condition of incremental sequence mining, an efficient incremental mining algorithm for discovering a sequential pattern in a WSN environment was proposed. The algorithm is comprised of two steps: obtain potential frequent sequence and count the support number, which is the same as the common mining algorithms. In the first step, the mining algorithm just needs to be carried out on the incremental database and the corresponding prefix projection database, avoiding mining on the original database repeatedly. In the second step, a data structure called a reticular sequence tree is designed to realize the fast-counting of the support number, reducing the times of scanning the original database greatly. Then, the whole efficiency of the proposed algorithm was increased dramatically. Intensive experiments were conducted on benchmarking datasets, synthetic datasets, and real monitoring datasets to analyze the NIA’s performances regarding time cost, sensitivity of factors, and space cost, which showed that the proposed algorithm is more efficient than the other methods.

Sensor data mining has become more and more important in real applications, for instance, in dam safety monitoring, where various kinds of sensing data are fed back in real time. If the key factors and inherent patterns related to the dam safety can be discovered from the data, this would be very meaningful for decision-making and management. The proposed algorithm NIA has been implemented on real monitoring data from a huge hydropower plant, and the discovered sequential patterns reflects the correlations between horizontal displacement and water level, which is quite useful for predicting the horizontal displacement of dam and analyzing the key positions that need to be paid more attention. Also, we are trying to use NIA to find out the relations between other different kinds of monitoring data.

For the future work, we will study how to use other technologies or theories to further improve PrefixSpan for more extensive sequence mining, and how to save the memory usage is worthy to be focused on as well.

## Figures and Tables

**Table 1:** Symbol Table.

*sdb*	Increment sequence database
UD	Updated database
OD	Sequences contained in dotted line area
DD	Combination of OD and old
|M|	The number of element in set M
$sup^{SDB}(\alpha )$	Support of α in *SDB*
$c^{SDB}(\alpha )$	Support number of α in *SDB*
$sup^{sdb}(\alpha )$	Support of α in *sdb*
$c^{sdb}(\alpha )$	Support number of α in *sdb*
$sup^{UD}(\alpha )$	Support of α in UD
$c^{UD}(\alpha )$	Support number of α in UD
old	The sequences contained in both *SDB* and *sdb*
new	The sequences contained in *sdb* that are different from *SDB*
$c^{OD}(\alpha )$	Support number of α in OD
$c^{DD}(\alpha )$	Support number of α in DD
$c^{old}(\alpha )$	Support number of α in old
$c^{new}(\alpha )$	Support number of α in new

**Table 2:** Sequence Database *SDB*.

*SID*	Sequence
1	<ad>
2	<b(ce)(ab)>
3	<(ab)(be)>
4	<dca>
5	<a>
6	<f(bfg)>

**Table 3:** Incremental Sequence Database *sdb*.

*SID*	Sequence
7	<(bd)>
8	<(bd)e>
9	<(ab)e(bf)>

**Table 4:** Updated Database UD.

*SID*	Sequence
1	<ad>
2	<b(ce)(ab)>
3	<(ab)(be)>
4	<dca>
5	<a>
6	<f(bfg)>
7	<(bd)>
8	<(bd)e>
9	<(ab)e(bf)>

**Table 5:** An Incremental Database of UD (*sdb*).

*SID*	Sequence
2	<d>
4	<h>
5	<(ad)>
8	<h>
10	<(bdfh)>
11	<ag>
12	<(bf)g>

**Table 6:** The New UD.

*SID*	Sequence
1	<ad>
2	$<b(ce)(ab)\underline{d}>$
3	<(ab)(be)>
4	$<dca\underline{h}>$
5	$<a(\underline{ad})>$
6	<f(bfg)>
7	<(bd)>
8	$<(bd)e\underline{h}>$
9	<(ab)e(bf)>
10	<(bdfh)>
11	<ag>
12	<(bf)g>

**Table 7:** OD (in which the *sid* is same as that used in old).

*SID*	Sequence
2	<b(ce)(ab)>
4	<dca>
5	<a>
8	<(bd)e>

**Table 8:** DD (which is the combination of OD and old).

*SID*	Sequence
2	$<b(ce)(ab)\underline{d}>$
4	$<dca\underline{h}>$
5	$<a(\underline{ad})>$
8	$<(bd)e\underline{h}>$

**Table 9:** An Example of Sequence Database.

*SID*	Sequence
1	<(30)(90)>
2	<(10,20)(30)(10,40,60,70)>
3	<(30,50,70,80)>
4	<(30)(30,40,70,80)(90)>
5	<(90)>

**Table 10:** The Support Number of the Prefixes with length=1.

**Prefix**	(10)	(20)	(30)	(40)	(50)	(60)	(70)	(80)	(90)
**Support Number**	1	1	4	2	1	1	3	2	3

**Table 11:** The Modified Sequence Database.

***SID***	**Sequence**
1	<(30)(90)>
2	<(30)(40,70)>
3	<(30,70,80)>
4	<(30)(30,40,70,80)(90)>
5	<(90)>

**Table 12:** CMAP of Length-1 Sequential Patterns.

Pattern	$cm_{i}\left(j\right)$	$cm_{s}\left(j\right)$
30	70, 80	40, 70, 90
40	70	-
70	80	-
80	-	-
90	-	-

**Table 13:** CMAP of Length-2 Sequential Patterns.

Pattern	$cm_{i}\left(j\right)$	$cm_{s}\left(j\right)$
(30)(40)	70	-
(30, 70)	80	-

**Table 14:** Frequent Sequential Patterns with minsup=25%.

	Frequent Sequential Pattern
length-1 sequential pattern	<(30)>,<(40)>,<(70)>,<(80)>,<(90)>
length-2 sequential pattern	<(30,70)>,<(30,80)>,<(30)(40)>,<(30)(70)>,<(30)(90)>
	<(40,70)>,<(70,80)>
length-3 sequential pattern	<(30)(40,70)>,<(30,70,80)>

**Table 15:** Experimental Parameters.

Parameter	Definition
|SDB|	Amount of sequences in *SDB*, unit: 1000
C	Average amount of items
T	Average amount of elements
S	Length of potential frequent sequence
I	Number of items in potential frequent itemset
N	Number of different elements in sequence database
$N_{I}$	Number of potential frequent itemsets
$N_{s}$	Number of potential frequent sequences
|UD|	Amount of sequences in UD, unit: 1000
$R_{sdb}$	Ratio of incremental database *sdb* to updated database UD
$R_{new}$	Ratio of new sequences to incremental database *sdb*
